# Targeting a Tau Kinase Cdk5, Cyclin-Dependent Kinase: A Blood-Based Diagnostic Marker and Therapeutic Earmark for Alzheimer’s Disease

**DOI:** 10.3390/biom15101365

**Published:** 2025-09-26

**Authors:** Sakshi Kumari, Abhinay Kumar Singh, Mukesh Kumar, Rashmita Pradhan, Abhijith R. Rao, Yudhishthir Yadav, Pramod Kumar, Partha Haldar, Punit Kaur, Sharmistha Dey

**Affiliations:** 1 Department of Biophysics, All India Institute of Medical Sciences, New Delhi 110029, India; sakshi@aiims.edu (S.K.); abhinaysingh@aiims.edu (A.K.S.); mukeshkumar@aiims.edu (M.K.); rashmita@aiims.edu (R.P.); yudhishthir@aiims.edu (Y.Y.); punitkaur@aiims.edu (P.K.); 2 Department of Geriatric Medicine, All India Institute of Medical Sciences, New Delhi 110029, India; abhijithrrao@aiims.edu (A.R.R.); pkumar1989@aiims.gov.in (P.K.); 3 Department of Community Medicine, All India Institute of Medical Sciences, New Delhi 110029, India; patha@aiims.edu

**Keywords:** Alzheimer’s Disease, Cdk5, Mcl1, SPR, blood-based biomarker, peptide inhibitor, SH-SY5Y

## Abstract

Protein kinases are important molecules of Alzheimer’s Disease (AD), driving neuronal demise and the emergence of the disease’s destructive hallmarks. Cdk5 has recently been highlighted as a key therapeutic target for AD. This study evaluated the expression levels of Cdk5 and Mcl1 (Cdk5’s substrate) in blood samples of 61 AD, 55 Mild Cognitive Impairment (MCI), and 57 Geriatric Controls (GC), and explored the in vitro inhibition of Cdk5. The serum levels of Cdk5 and Mcl1 were measured by Surface Plasmon Resonance (SPR) and verified by Western blot and RT-PCR. Molecular modeling and simulation studies were used to identify a potent hit targeting Cdk5 and validated by binding studies using SPR. The peptide rescue effect was analyzed by MTT assay in the AD cellular model. SPR analysis revealed a significant change in Cdk5 and Mcl1 levels in the serum samples of AD and MCI compared to GC. Results were validated by Western blot and RT-PCR. Binary logistic regression analysis revealed that the concentration of both Cdk5 and Mcl1 was independently associated with disease after adjusting for certain parameters. ROC analysis established an optimum diagnostic cutoff value for Cdk5 [24.97 ng/µL (AUC-0.90)] and Mcl1 [23.08 ng/µL (AUC-0.94)] with high sensitivity and specificity. The peptide YCWS strongly binds to Cdk5′s ATP binding site, confirmed by molecular modeling and SPR. In the AD cellular model, peptide YCWS rescued neurotoxicity, increased Mcl1 levels, and reduced destructive hallmarks by inhibiting Cdk5. It can be concluded that Cdk5 is a promising molecule as a circulatory biomarker for the diagnosis of the early stages of AD, and its peptide inhibitor YCWS is a potential therapeutic agent.

## 1. Introduction

The pathological characteristics of Alzheimer’s Disease (AD) are well-established and serve as a groundwork for clinical assessment and disease detection. Despite that, effective prevention and treatment strategies remain elusive. One of the primary challenges in treating AD is the late-stage diagnosis, which often occurs after significant molecular changes have already taken place at the early onset of the disease. While early-onset symptoms do not become apparent until much later. A lot of research is going on to locate the early events that initiate the well-known hallmarks (amyloid plaque and tau tangles) of the disease. Inflammation and oxidative pathways are two major episodes in the life cycle that control many biological functions. Cdk5 (Cyclin-Dependent Kinase 5) is one of the unique kinases that is involved in inflammation and is associated with several synaptic functions. Cdk5 is a key enzyme involved in the pathological phosphorylation of Tau protein, a major component of neurofibrillary tangles. Elevated levels of Cdk5 activity led to excessive phosphorylation of the Tau protein, which contributes to the formation of neurotoxic aggregates [1]. Cdk5 also triggers neurotoxic Aβ-amyloid formation along with hyperphosphorylation of tau protein [2,3].

Cdk5 is of prime significance and is undoubtedly a biomolecule of utmost importance for neurons and is acknowledged to play an integral role in maintaining not only the neuronal structure but also executing a plethora of functions within the neurons. Cdk5 is widely expressed in mammalian tissues and organs, although it exhibits high activity only in the brain tissue [4,5,6] specifically due to the expression of its two well-known activators, p35 and p39 [7,8]. In the different developmental stages of the rat nervous system, p35 has been reported to be mainly distributed in the cerebral cortex, and p39 in the cerebellum region [7,8]. Calpain-mediated cleavage of p35 yields a more stable p25 form (half-life up to 5–10 times longer than that of p35), leading to the prolonged activation and mislocalization of Cdk5, resulting in hyperphosphorylation of tau, disruption of the cytoskeleton, and ultimately leading to neuronal death [9,10,11,12,13].

The countless roles of Cdk5 in AD make it a potential target for curing the disease [14,15,16,17,18]. Cdk5 is known to have a unique direct substrate, Mcl1, which makes it different from other kinases [19]. Mcl1, Myeloid Cell Leukemia 1, is an anti-apoptotic protein and is known to be involved in various biological processes. Several studies have reported the importance of Mcl1 in mitochondrial functioning, PINK1-PRKN/PARK2 signaling, and autophagy. Mcl1 is also known to have a role in embryonic development, formation of synapses, regulation of lifespan, and various cellular processes, suggesting its importance in proper physiological functioning. Degradation of Mcl1 is regulated by the posttranslational modifications in the PEST domain by various kinases, i.e., Cdk5, GSK3β, JNK, p38, CK2 and CDK1. Although multiple kinases are involved in these modifications, Cdk5 has been identified as a direct substrate of Mcl1.

Reduced activity and hyperactivity of Cdk5 are equally neurotoxic, leading to various neurological disorders. Overexpression of Cdk5 during any injury or inflammation alters the phosphorylation state of cytosolic and cytoskeletal proteins, which are associated with AD [20,21].

Cdk5 is currently one of the lead molecules for the therapeutic target against neurodegenerative disease [22,23,24]. Some ATP competitive inhibitors, i.e., roscovitine, hymenialdisine, olomousine, alosine A, indirubin, dinaciclib, and ATP non-competitive small peptide inhibitors, are reported to inhibit Cdk5 [25,26,27,28,29,30,31]. However, the lack of selectivity is a major drawback. Other inhibitors are known to target the Cdk5/p25 kinase complex, as p25 activates Cdk5. The level of p25 has also been reported to be higher in the AD brain [12,13,32,33]. Unfortunately, many Cdk5 inhibitors lack specificity due to their shared binding site with other kinases, which often leads to off-target effects. Despite ongoing efforts, none of the molecules have been reported to selectively inhibit Cdk5, thus requiring more effective drug development to prevent and treat the disease.

The currently available diagnosis methods include cerebrospinal fluid (CSF) biomarkers (amyloid-β, total tau, and phosphorylated tau) and positron emission tomography (PET) imaging; both are invasive, expensive, or not readily accessible in many clinical settings. Also, these can only be diagnosed at the later stages of the disease.

Inflammation is the early event in the pathogenesis of disease, resulting in the hyperactivation of Cdk5. Thus, Cdk5/Mcl1 may be used as significant blood-based biomarkers for the detection of disease at an early stage and can facilitate recruitment into clinical trials.

This study aims to determine whether higher or lower expression of Cdk5 levels in the blood of AD and Mild Cognitive Impairment (MCI) patients is associated with an increased risk of cognitive decline by correlating with Mcl1 level.

This study also aimed to reduce the overexpression of Cdk5, thereby increasing the Mcl1 levels and potentially mitigating Aβ-amyloid and hyperphosphorylated tau formation in disease pathology in an AD cellular model by a designed peptide inhibitor targeting Cdk5.

## 2. Methods

### 2.1. Ethics

The study protocol was approved in accordance with the Declaration of Helsinki by the Institute Ethics Committee for Postgraduate Research, All India Institute of Medical Sciences (AIIMS), New Delhi, India (Ref No. IEC-39/07.02.2020, RP-19/2020). Both oral and written consent were obtained from the individuals participating in the study and from their attendants.

#### 2.1.1. Patient Recruitment Criteria

During the period (20 January 2021 to 3 July 2024), 659 individuals visited the Memory Clinic of the Department of Geriatric Medicine, AIIMS, New Delhi, and were clinically assessed. No formal power calculation was conducted prior to recruitment, as this was a new and exploratory study aimed at assessing differences in Cdk5 and Mcl1 levels across AD, MCI, and control groups. In this study, 173 subjects were enrolled, 61 with AD and 55 with MCI, along with 57 individuals who were recruited from the Geriatric Medicine OPD as Geriatric Control (GC). All subjects diagnosed with MCI met Petersen’s 2011 criteria as mentioned earlier [34]. Disease was diagnosed mainly via two steps; firstly, the subjects with cognitive impairment were screened for the Hindi Mini-Mental State Examination (HMSE) and Addenbrooke’s Cognitive Examination (ACE III), and next they were examined for MRI, tau PET, FDG PET, and other tests like TSH, folate, and vitamin B12. The depression level and functionality in study subjects were assessed using the Geriatric Depression Scale (GDS) and Instrumental Activities of Daily Living (IADL-EDR) scale. Subjects with AD and MCI were aged over 60 years, had cognitive impairment, and had consented to participate in the study. Subjects with neurodegenerative disorders other than AD and MCI (i.e., Parkinson’s Disease, Lewy body dementia, multiple sclerosis) or having chronic inflammatory conditions of COPD, heart failure, and rheumatoid arthritis were excluded from the study. GC subjects were >60 y, with no clinically apparent cognitive impairment, and consented to enroll in the study.

#### 2.1.2. Collection and Processing of Blood Sample

A 5 mL peripheral blood sample was obtained from each participant and aliquoted into two sterile collection tubes: a plain tube for serum separation and an EDTA-coated tube for mRNA isolation. The blood sample in a plain tube was allowed to clot at room temperature for 30–40 min and then centrifuged at 3000 rpm/845 g for 10 min. The serum was collected immediately and stored at −80 °C for further use.

#### 2.1.3. Evaluation of Protein Level in Serum Samples by Surface Plasmon Resonance (SPR)

The protein concentration in the serum sample was evaluated using the Surface Plasmon Resonance (SPR) technology. All experiments were performed at 25 °C using the BIAcore-3000 (GE Healthcare, Uppsala, Sweden), a biosensor-based system for real-time specific interaction analysis. The following primary antibodies, mouse IgG against human Cdk5 (Santa Cruz Biotechnology Inc., Dallas, TX, USA, cat. no. Sc6247) and rabbit IgG against human Mcl1 (Cell Signaling Technologies, cat. no. CST-45725), were immobilized on the different flow cells of a CM5 sensor chip employing an amine coupling kit, following the manufacturer’s protocol (GE Healthcare, Uppsala, Sweden). The antibody immobilization was performed as previously reported [35]. A recombinant clone containing the Cdk5 gene in the pET-28b (+) expression vector with a 6 His tag was obtained from GenScript, Piscataway, NJ, USA. The Mcl1 gene was cloned in the pJET vector, using the gene-specific primers (Forward-5′-GGA TCC ATG TTT GGC CTC AAA AGA-3′ and Reverse-5′-CCG AAG CTT CTA TCT TAT TAG ATA TGC C-3′). The recombinant clones were further transformed into BL21 cells, expressed, and the protein was purified using affinity chromatography and further characterized by Western blot.

Different concentrations of purified recombinant human Cdk5 and Mcl1 proteins were passed over the respective flow cells, and corresponding response units (RU) were noted. The standard curve was prepared by plotting different concentrations of pure proteins versus the corresponding RU (Figure S1). Serum samples were diluted (1:79) with HBS-EP buffer and passed over the respective flow cell. The RU for each sample was noted, and the concentration of Cdk5 and Mcl1 in the serum sample was derived from their respective standard curves.

#### 2.1.4. Validation of Proteins in Serum Sample by Western Blot

Randomly, three samples from each study group were selected for the experiment. Albumin depletion of the samples was performed as per the manufacturer’s instructions (cat. no. 786252, GBiosciences, St. Louis, MO, USA). The concentration of total protein in the serum sample was measured using the bicinchoninic acid (BCA) assay (cat. no. PRTD1). Samples were prepared using the 6× protein loading dye [9% β-mercaptoethanol, 0.03% bromophenol blue, 48% glycerol, 6% SDS, 375 mM Tris HCL (1 M, pH 6.8)] and incubated at 95 °C for 15 min. Samples (4 mg/lane) were fractionated by 10% sodium dodecyl sulfate–polyacrylamide gel electrophoresis (SDS-PAGE) and transferred to polyvinylidene difluoride (PVDF, cat. no. IPVH00010) membrane (MDI Membrane Technologies, Ambala Cantt, India). The membrane was blocked with 5% non-fatty dry milk (NFM) for 2 h, and then probed with primary antibodies—Cdk5 (1:400) and Mcl1 (1:1000) diluted in TBS-T (20 mM Tris pH 7.5, 500 mM NaCl, 0.05% Tween 20)—overnight at 4 °C. The membrane was further incubated with HRP-conjugated secondary antibodies: goat anti-mouse IgG (1:4000) (Gen Script, Piscataway, NJ, USA) for Cdk5 and goat anti-rabbit IgG (1:4000) (Gen Script, Piscataway, NJ, USA) for Mcl1 at room temperature for 1 h. Western blot bands were captured using the Enhanced Chemiluminescent System (Bio-Rad, Bengaluru, India), and the signal was quantified via my Image Analysis software Celleste 6 (Thermo Scientific, Waltham, MA, USA).

#### 2.1.5. Isolation of mRNA from Blood Sample of AD, MCI and GC Subjects Followed by Quantitative Real-Time PCR

A blood sample collected in an EDTA-coated tube was used for the isolation of mRNA, using the Ribozol method as per the manufacturer’s instructions. RNA quantity and quality were controlled by spectrophotometric analysis and gel electrophoresis. RNA (1 μg) was reverse transcribed into cDNA using the RevertAid First Strand cDNA Synthesis Kit (cat. no. K1622, Thermo-fisher, Waltham, MA, USA) as per the protocol provided by the manufacturer. cDNA (100 ng) was added to detect the amplification-specific PCR products of Cdk5, Mcl1, and GAPDH in Brilliant III Ultra-Fast SYBR Green QPCR Master Mix (cat. no. 600882). All reactions were conducted in triplicate using the following primers: Cdk5 forward primer—5′-AAT GAC TGG GAG GAG AGA GGG AG-3′ and reverse primer—5′-TTC ACG GCG TGC ATA CTC AG-3′ [36]; Mcl1 forward primer—5′-AGG CTG GGA TGG GTT TGT G-3′ and reverse primer—5′-CAC ATT CCT GAT GCC ACC TTC T-3′ [37]; GAPDH forward primer—5′-ACC ACA GTC CAT GCC ATC AC-3′ and reverse primer—5′-TCC ACC ACC CTG TTG CTG TA-3′. The reaction comprises an initial denaturation step at 95 °C for 10 min, followed by 40 cycles of denaturation at 95 °C for 30 s, annealing at different temperatures for 1 min, and extension at 72 °C for 1 min. The different annealing temperatures used were 58 °C for Cdk5 and GAPDH and 60 °C for Mcl1. All reactions were ended with a melting curve analysis. Results were normalized against the internal control (GAPDH) using the δ-δ CT method [38].

### 2.2. Molecular Docking

The structure of the Cdk5 complex with its known inhibitor roscovitine (PDB ID: 1UNL) was accessed on 2 November 2024 from the Protein Data Bank (www.rcsb.org) and prepared using the GLIDE v9.1 docking tool. This preparation involved removing water molecules, adjusting protonation states, and adding missing residues and hydrogen atoms, followed by energy minimization under the default settings of the OPLS_2005 force field. The tetrapeptide library was processed through the LigPrep wizard in the Schrödinger suite’s Glide module, generating up to 32 tautomers for each tetrapeptide within a pH range of 7.0 ± 2.0. Roscovitine was also prepared to validate the docking protocol, confirming the methodology’s ability to reproduce known binding orientations and affinities. A 20 Å grid was generated around specific active site residues using the Glide grid preparation wizard. In silico screening and docking were subsequently performed with a rigid receptor grid, allowing flexibility in the tetrapeptides.

#### 2.2.1. Molecular Dynamics Simulations

Molecular dynamics (MD) simulations were conducted for the top five peptides—YCWS, YWCS, VWCS, DHYW, FWYS—to form complexes with the Cdk5 protein using Desmond software with the TIP3P water model. The prepared protein–peptide complexes from the docking studies were imported into the Desmond interface, and the system was solvated with TIP3P explicit water, compatible with the OPLS-AA force field for simulating biological conditions. An orthorhombic simulation box was generated, providing a 10 Å buffer to prevent boundary artifacts. To neutralize the system, counterions (Na^+^ or Cl^−^) were added, and a 0.15 M NaCl concentration was introduced to mimic physiological conditions. The system then underwent energy minimization and equilibration. MD simulations were run for 100 ns with a 2 fs timestep, recording trajectory frames every 10 ps. Post-simulation analysis involved examining root mean square deviation (RMSD), root mean square fluctuation (RMSF), hydrogen bonding, and binding interactions to evaluate the stability and dynamic behavior of the complexes.

#### 2.2.2. Binding Free Energy Evaluation Using Prime-MMGBSA

The Molecular Mechanics Generalized Born Surface Area (MMGBSA) method was applied to snapshots taken from the last 30 ns of a stabilized trajectory, offering a more accurate assessment of ligand binding affinity. For each snapshot, energies were computed using the OPLS-AA force field, and solvation energy was calculated using the Generalized Born (GB) model. Binding free energy (ΔG) was derived by calculating the energy difference between the protein–ligand complex and the individual protein and ligand.

### 2.3. Peptide Synthesis

The peptide was synthesized by Peptide Synthesizer PS3 (Protein Technology, Tucson, AZ, USA) using the solid-phase peptide synthesis employing N-terminal protector Fmoc and Wang resin chemistry. Peptide was synthesized from C terminal to N terminal using Dimethylformamide (DMF) as a solvent, 20% piperidine as a deprotector, and 2-(1H-Benzotriazole-1-yl)-1,1,3,3 tetramethyluronium hexafluorophosphate (HBTU) and N-Methylmorpholine (NMM) as an activator. Serine (S) amino acid attached to Wang resin (Chem Impex, Wood Dale, IL, USA) was taken, and after deprotection, it was coupled with the next activated amino acid, i.e., tryptophan (W), and the process continues till the synthesis of tetrapeptide is complete. Wang resin was finally cleaved using trifluoroacetic acid (TFA), and the peptide was precipitated from chilled diethyl ether. The peptide was dissolved in 10% acetic acid and lyophilized for further use.

The Aβ_25–35_ peptide was also synthesized in a similar manner, starting with methionine (M) attached to the wang resin at the C-terminal end.

### 2.4. In Vitro Studies

#### 2.4.1. Binding Study of Peptide by SPR

The recombinant Cdk5 protein was immobilized on the Ni-NTA sensor chip by passing NiCl2 solution and the protein. Different concentrations of peptide YCWS (0.4 µM, 0.6 µM, and 1 µM) were passed over it, and changes in RU were noted. The association constant (KA) and dissociation constant (KD) values were calculated using the BIA evaluation 3.0 software.

#### 2.4.2. Cells and Treatment

SH-SY5Y and HEK 293 cells were purchased from NCCS, Pune, India, and were maintained in Ham’s F12 (cat. no. 21700075) and Dulbecco’s Modified Eagle Medium (DMEM, cat. no. 12800017) nutrient media (Gibco, Waltham, MA, USA), respectively, supplemented with 10% (*v*/*v*) Fetal Bovine Serum (FBS, cat. no. 10270106) and 1% antibiotic–antimycotic solution (cat. no. CC4003010L, CELL clone, New Delhi, India). The SH-SY5Y cells used in the experiment were of passage number 42–46. Cells were maintained as a monolayer culture at 37 °C with 5% CO_2_ and 95% relative humidity. Potential contamination in all cells was assessed through STR profiling (DNA Forensic Laboratory Private Limited, New Delhi, India).

#### 2.4.3. Cell Cytotoxicity Assay in HEK-293 Cells

The peptide’s effect was initially assessed using the normal HEK 293 cell line, and the cell viability was determined by the 3-(4,5-dimethylthiazol-2-yl)-2,5-diphenyltetrazolium bromide (MTT) (cat. no. T0793) reduction assay. Cells were seeded in 96-well plates at a density of 5 × 10^3^ cells per 100 μL per well. Cells were cultured for 24 h and then treated with different concentrations of peptide, i.e., 10 µM, 20 µM, and 50 µM, for different time intervals (24 h, 48 h, and 72 h). At the end of treatment, 10 μL MTT (5 mg/mL) was added in each well and incubated for 4 h at 37 °C. The media was aspirated, and 100 μL of DMSO was added to dissolve formazan crystals and incubated for 30 min at 37 °C. The absorbance value at 572 nm was measured using a microplate reader. Experiments were performed in triplicate wells to ensure reproducibility.

#### 2.4.4. Inhibitor Peptide Mediated Rescue Effect in SH-SY5Y Cells

Aggregated Aβ_25–35_ was employed to induce neurotoxicity in SH-SY5Y cells due to its ability to replicate the physiological and biological characteristics of Aβ-amyloid, known to mediate neurotoxic effects and contribute to neuronal cell damage. The effect of peptide on neurotoxicity caused by aggregated Aβ_25–35_ was assessed via MTT assay. Cells were seeded in 96-well plates at a density of 1 × 10^4^ cells per 100 μL per well. Cells were cultured for 24 h and then treated with 20 µM retinoic acid for differentiation. The culture medium was routinely replaced every alternate day for a period of seven days. A stock solution of 20 mM Aβ_25–35_ was prepared by dissolving 2.12 mg of synthesized Aβ_25–35_ in 100 µL of 1× PBS and allowed to aggregate at 37 °C for 7 days. Cells were treated with 20 µM aggregated Aβ_25–35_, along with different concentrations of the peptide YCWS (2 µM, 5 µM, 10 µM, 15 µM, and 20 µM) for 24 h, 48 h, and 72 h. After completion of the treatment duration, cell viability was determined using the MTT assay as described above.

Similarly, cells were seeded in a T25 flask (1 × 10^6^ cells) and a 35 mm Petri dish (0.2 × 10^6^ cells) in six different groups, i.e., for control, vehicle control, aggregated Aβ_25–35_ treated, and the 3 treatment groups for cell lysate preparation and mRNA isolation. Cells were collected and lysed using RIPA buffer (10 mM Tris-HCl pH 8.0, 140 mM NaCl, 1 mM EDTA pH 8.0, 0.1% sodium deoxycholate, 1% Triton X-100, 0.1% SDS, 0.1 mM ethylene glycol tetra acetic acid pH 8.0, 1 mM protease inhibitor, 1 mM phenyl-methane sulfonyl fluoride) to prepare the cell lysate. Isolation of mRNA and synthesis of cDNA were performed as mentioned above. cDNA was used as a template to amplify Cdk5-specific PCR products using Brilliant III Ultra-Fast SYBR Green QPCR Master Mix. The reaction conditions and the primers are mentioned above. Finally, results were normalized against the internal control (GAPDH) using the δ-δ CT method.

#### 2.4.5. Expression of Cdk5, Mcl1, Aβ-Amyloid, Tau, and *p*-Tau Proteins by Western Blotting

The concentration of total protein in cell lysate was measured using the bicinchoninic acid (BCA) assay. Samples were prepared using the 6× protein loading dye and incubated at 95 °C for 15 min. Equal concentrations of cell lysate (50 µg/lane) were fractionated by 10% SDS-PAGE and transferred to PVDF membrane. The membrane was blocked with 5% NFM for 2 h and then probed with their respective primary antibodies: Cdk5 (1:400) and Mcl1 (1:1000), mouse IgG anti-β-amyloid (1:200) (Sc28365), mouse IgG anti-Tau (Tau-46) (1:200) (Santa Cruz Biotechnology Inc., Dallas, TX, USA, Sc32274), rabbit IgG anti-phospho-Tau (Thr181) (1:350) (Cell Signaling Technology, Inc., Danvers, MA, USA, DAF4G), and rabbit antibody IgG anti-β-actin (1:1000) (Cell Signaling Technology, Inc., Danvers, MA, USA). The membranes were incubated with the secondary anti-rabbit and anti-mouse (cat. no. Sc2004, Sc2005) HRP-conjugated antibody (1:4000) at room temperature for 1 h. Western blot bands were captured using the Enhanced Chemiluminescent System, and the signal was quantified via my Image Analysis software. Protein expression in each group was normalized to the internal control using β-actin.

### 2.5. Statistical Analysis

Data were managed using a Microsoft Excel database. Statistical analyses were performed using GraphPad Prism 8 and STATA version 14. Results are presented as mean ± standard deviation (SD) for continuous variables and as frequencies (percentages) for categorical variables. Comparisons of continuous variables among groups were conducted using one-way ANOVA, followed by Bonferroni post hoc tests. The chi-square test was applied to assess differences in categorical variables. Correlations between continuous variables (protein concentration and HMSE or ACE-III scores) were evaluated using the Pearson correlation coefficient. A *p*-value < 0.05 was considered statistically significant.

## 3. Results

### 3.1. Demographic and Clinical Data of Study Participants

The demographic and clinical data of 173 subjects are illustrated in Supplementary Table S1. The HMSE (*p*-value ≤ 0.0001) and ACE III (*p*-value ≤ 0.0001) scores were significantly lower in AD as compared to the MCI and GC. The percentage of male subjects was higher than that of the females in all three study groups. Depending upon the age, the subjects were divided into three different categories, i.e., 60–65 y, 66–75 y, and ≥76 y. The majority of the subjects belong to the 66–75 y category, i.e., 62.30% in AD, 69.09% in MCI, and 57.89% in GC (*p* = 0.009). A high literacy rate was observed in all three groups of the study. Most of the enrolled subjects were from the urban population. Disease duration of AD and MCI was mostly above 2 y. Few of the subjects, i.e., 22.95% in AD and 25.45% in MCI, show a family history of the disease. There was a significant difference in the mean ± SD of the clinical scores, i.e., CDI (*p* ≤ 0.0001), HMSE (*p* ≤ 0.0001), ACE III (*p* ≤ 0.0001), and GDS (*p* = 0.0128), between the study groups.

#### 3.1.1. Evaluation of Cdk5 and Mcl1 in the Serum Sample of Study Groups by SPR

**Figure 1:**
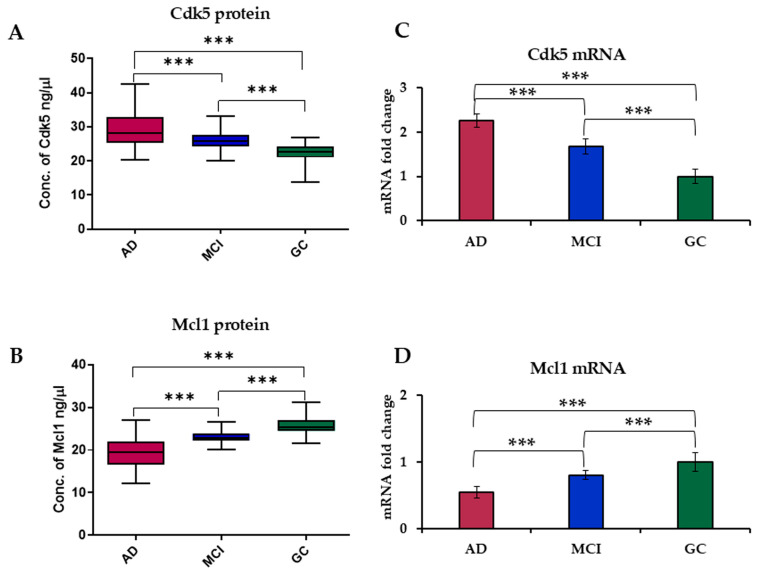
Box plot of serum level of (**A**) Cdk5 and (**B**) Mcl1 in AD, MCI and GC. mRNA expression levels of (**C**) Cdk5 and (**D**) Mcl1 in blood samples of AD, MCI and GC subjects using RT-PCR. Cyclin-Dependent Kinase 5 (Cdk5), Myeloid Cell Leukemia 1 (Mcl1), Alzheimer’s Disease (AD), Mild Cognitive Impairment (MCI) and Geriatric Control (GC). Experiments were performed in triplicate, and data is presented as mean ± SD (serum level) and mean ± SEM (mRNA level) [*n* = 61 (AD), 55 (MCI) and 57 (GC)]. One-way ANOVA followed by post hoc comparison using the Bonferroni test was used to compare between groups. Significantly different at; *** *p* < 0.0001.

The concentration of Cdk5 (mean ± SD) in serum level was significantly higher (*p* < 0.0001) in AD (29.42 ± 5.49) as compared to MCI (26.18 ± 2.79) and GC (22.52 ± 2.26) subjects. While the level of Mcl1 was found to be significantly lower (*p* < 0.0001) in AD (19.51 ± 3.44) as compared to MCI (23.10 ± 1.26) and GC (25.74 ± 2.00) (Figure 1A,B), the concentration of Cdk5 (ng/μL) and Mcl1 (ng/μL) in serum with different attributes is illustrated in Supplementary Tables S2 and S3.

#### 3.1.2. mRNA Expression Level of Cdk5 and Mcl1 in the Blood Sample of Subjects

Expression levels of Cdk5 and Mcl1 mRNA were determined using RT-PCR. Analysis revealed a 2.25-fold increase in the expression level of Cdk5 in AD and a 1.67-fold increase in MCI as compared to GC subjects. While in the case of Mcl1, there was a 0.54-fold decrease in AD and a 0.81-fold decrease in MCI as compared to GC subjects (Figure 1C,D).

**Figure 2:**
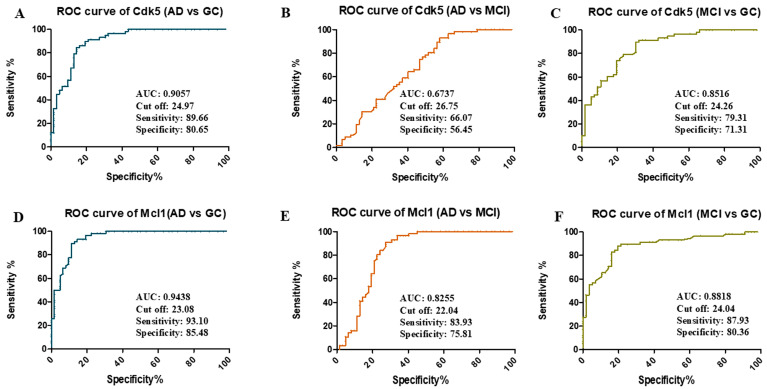
Receiver-operating characteristic (ROC) curve of Cdk5: (**A**) AD vs. GC, (**B**) AD vs. MCI, (**C**) MCI vs. GC, and Mcl1 (**D**) AD vs. GC, (**E**) AD vs. MCI, (**F**) MCI vs. GC, determining area under curve (AUC), cutoff values, sensitivity and specificity. Alzheimer’s Disease (AD = 61), Mild Cognitive Impairment (MCI = 55), Geriatric Control (GC = 57), Cyclin-Dependent Kinase 5 (Cdk5), and Myeloid Cell Leukemia 1 (Mcl1).

The receiver operating characteristic (ROC) curve was generated on the basis of SPR data to analyze the potential of Cdk5 and Mcl1 as biomarkers for the diagnosis of the disease (Figure 2). From ROC analysis and Youden’s index, the area under curve (AUC) for Cdk5 was 0.90 for differentiating AD vs. GC (24.97 ng/µL cutoff, 89.66% sensitivity, and 80.65% specificity), 0.67 AUC for differentiating AD vs. MCI (26.75 ng/µL cutoff, 66.07% sensitivity, and 56.45% specificity), and 0.85 AUC for differentiating MCI vs. GC (24.26 ng/µL cutoff, 79.31% sensitivity, and 71.31% specificity). In the case of Mcl1, the AUC was 0.94 for differentiating AD vs. GC (23.08 ng/µL cutoff, 93.10% sensitivity and 85.48% specificity), 0.82 AUC for differentiating AD vs. MCI (22.04 ng/µL cutoff, 83.93% sensitivity and 75.81% specificity), and 0.88 AUC for differentiating MCI vs. GC (24.04 ng/µL cutoff, 87.93% sensitivity, and 80.36% specificity) (Supplementary Table S4).

**Figure 3:**
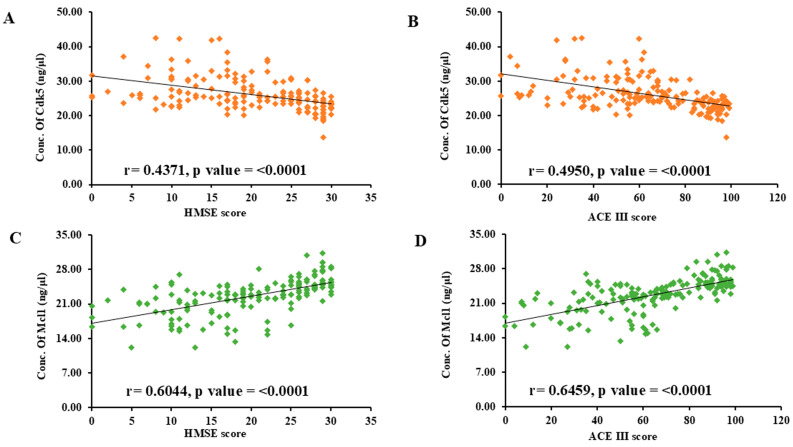
Scatter diagram showing correlation between serum protein concentration (ng/μL) and clinical scores: (**A**) Cdk5 vs. HMSE score, (**B**) Cdk5 vs. ACE III score, (**C**) Mcl1 vs. HMSE score, (**D**) Mcl1 vs. ACE III score by Pearson’s correlation analysis. Cyclin-Dependent Kinase 5 (Cdk5), Myeloid Cell Leukemia 1 (Mcl1), Hindi Mini-Mental State Examination (HMSE) and Addenbrooke’s Cognitive Examination (ACE III).

Further, Pearson correlation analysis was performed to correlate the protein concentration with clinical scores. The lower HMSE and ACE III scores were significantly associated with the higher concentration of Cdk5 and the lower concentration of Mcl1. For the correlation between Cdk5 and HMSE, r = 0.4371 and *p* < 0.0001; for Cdk5 and ACE III, r = 0.4950 and *p* < 0.0001. For the correlation between Mcl1 and HMSE, r = 0.6044 and *p* < 0.0001; for Mcl1 and ACE III, r = 0.6459 and *p* < 0.0001 (Figure 3). To study the strength of association between biomarker and cognition (MCI and AD), logistic regression was used. Further, the regression model was adjusted for age, sex, education, family history of AD, hypertension, diabetes, and coronary artery disease. The results are presented as odds ratio (OR) and 95% confidence interval (CI).

#### 3.1.3. Western Blot of Cdk5 and Mcl1 in Serum Sample

Western blot also showed a significant increase in expression of Cdk5 in AD and MCI subjects as compared to GC, while Mcl1 levels showed an inverse correlation with the disease severity. It was found to be significantly lower in AD as compared to MCI and GC subjects (Figure S2).

### 3.2. Molecular Docking

Molecular docking studies were conducted using the Glide module in Maestro (Schrödinger). Screening was carried out in standard precision (SP) mode. First, control ligand (roscovitine) docking was performed to validate and optimize the docking protocol, which was subsequently applied to all five selected peptides: YCWS, YWCS, VWCS, DHYW, and FWYS.

**Figure 4:**
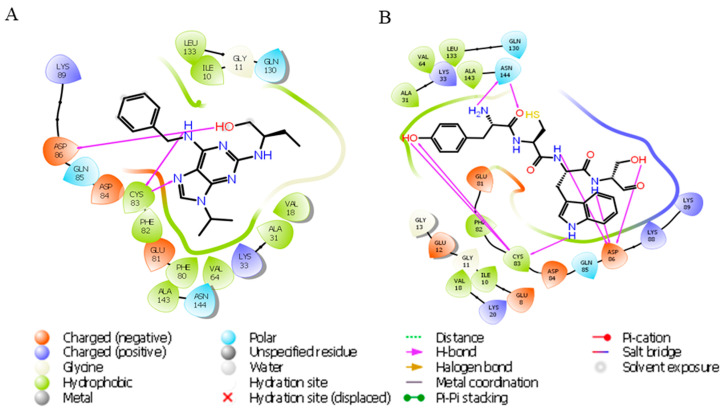
Ligand interaction diagram of roscovitine and YCWS showing hydrogen bonding (magenta line) with Cdk5 (PDB: IUNL) protein. (**A**) Roscovitine forms two hydrogen bonds with Cys83 and one with Asp86. (**B**) YCWS forms two hydrogen bonds with Asn144 and three each with Asp86 and Cys83. All representations are predicted by Glide SP docking in the Schrödinger suite.

In the previous docking study of the Cdk5–roscovitine complex, it was found that it forms hydrogen bonds with Cys83 and Asp86 of Cdk5. Additionally, hydrophobic interactions significantly contribute to complex stability, involving residues Phe80, Phe82, Ile10, Ala31, Val18, Val64, Leu133, and Ala143. These interactions collectively enhanced the binding affinity and stability of the ligand within the binding site, providing insights into key residues involved in ligand recognition and stabilization (Figure 4A).

In our docking study of the Cdk5–YCWS complex, similar hydrogen bonds were formed. A total of eight hydrogen bonds were identified: two with Asn144 [two distinct hydrogen bonds with the NH and carbonyl groups of Tyr (Y)], and three each with Cys83 [two with the -OH group of Tyr (Y) and one with the indole -NH group of Trp (W)] and Asp86 [one with the peptide N of both Cys (C) and Ser (S) and one with the -OH group of Ser (S)]. Additional contributing residues include Asp84, Gln130, Lys33, Glu12, Ile10, and Asp92, supporting the stability of the complex through multiple contacts. The polar contacts were observed with residues such as Lys20, Lys33, Lys88, Lys89, Asp84, Glu8, Glu12, Glu81, and Gln130. Hydrophobic contacts were stabilized by residues Ala31, Ala143, Ile10, Val16, Val18, Phe82, and Leu133. These interactions collectively contribute to the stable binding conformation of the peptide YCWS within the Cdk5 binding pocket (Figure 4B).

In the YWCS docked complex, a total of five hydrogen bonds were observed, i.e., with Glu81, Asn131, Glu8, Asn144, and Asp86 (also forms a salt bridge with the ammonium ion of Tyr). Furthermore, several hydrophobic contacts involving Ile10, Val18, Ala31, Val64, Phe80, Phe82, Cys83, Leu133, and Leu164 contribute to the stability of the YWCS complex within the binding pocket. These combined interactions suggest a stable binding conformation in the pocket (Figure S3A).

The VWCS docked complex forms two hydrogen bonds with Asp86 and one with Cys83, Asn144, and Asn131 each. Several hydrophobic interactions also contributed to the stability of the complex, involving residues such as Ala31, Ala143, Ile10, Phe80, Phe82, Leu133, and Gln130. These interactions collectively stabilized the VWCS peptide complex, ensuring a stable conformation within the binding pocket (Figure S3B).

The DHYW docked complex formed three hydrogen bonds with Asp86 and one with Cys83, Gln130 and Lys89 each. Beyond hydrogen bonding, the DHYW peptide was further stabilized by π-cation interactions between Lys33 and the phenyl ring of Tyr (Y) and by a π-π interaction with Phe80, reinforcing its position in the binding pocket. Asp86 and Lys89 also showed salt bridge interaction with the amino group and the -OH group of the Asp (D). Numerous hydrophobic interactions with residues Ala143, Ala31, Ile10, Leu133, Val18, Phe82, Gln85, and Asn131 contributed to the overall stability of the ligand–protein complex (Figure S3C).

The FWYS docked complex formed three hydrogen bonds with Asp86 and one with Glu12, Glu8, and Ile10 each. Beyond these hydrogen bonds, several hydrophobic interactions enhanced the complex’s stability, involving residues Val18, Leu133, Val64, Ala31, Phe80, and Phe82. Together, these interactions supported a stable binding conformation for the FWYS peptide (Figure S3D).

#### 3.2.1. Molecular Dynamic Simulations

**Figure 5:**
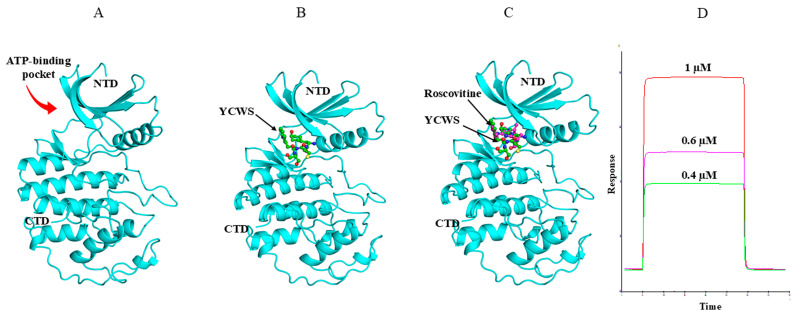
Binding mode of YCWS and roscovitine with CDK5 (PDB: IUNL). (**A**) Cdk5 is represented in cyan color. (**B**,**C**) The ATP-binding pocket between the N and C terminal lobes of the kinase is occupied by YCWS (green) in a similar way as it is occupied by roscovitine (magenta), as observed by the superimposition of roscovitine and peptide YCWS. The main structural elements of the kinase have been labeled. The figures were prepared using PYMOL software 3.1. (**D**) Sensorgram showing binding of different concentrations (0.4 µM, 0.6 µM and 1 µM) of YCWS on Ni–NTA sensor chip immobilized with Cdk5 protein by SPR analysis.

Molecular dynamics (MD) simulations provided crucial validation and refinement of the interactions between Cdk5 and the YCWS ligand, offering a dynamic, time-resolved view of the complex in an explicit solvent environment. This analysis underscored the importance of key residues, i.e., Gln130, Lys33, Glu12, Ile10, and Asp92, that contributed significantly to complex stability. Additional ionic interactions involving Lys33, Thr14, and Asn144 further reinforced the binding affinity and stability of the Cdk5–YCWS complex. The stability of the protein–ligand complex was substantiated by RMSD analysis (F), which exhibited minimal fluctuation within a narrow 1.15 Å range over the simulation, indicating that the complex maintained a stable structural configuration. YWCS also showed good peptide stability, with RMSD values mostly between 1.0 and 1.5 Å, though with slightly more fluctuations than YCWS. Furthermore, RMSF analysis revealed restricted fluctuations within binding-site residues, supporting the stable binding conformation of the Cdk5–YCWS complex. This combined docking and MD approach demonstrated YCWS’s unique binding pattern (Figures S4 and S5) and superior interaction profile compared to the control molecule, highlighting MD simulations’ critical role in refining docking predictions and capturing conformational adjustments (Figure 5A–C).

The peptide (YCWS) created an excellent binding network, mostly by latching onto Asp86 almost all the time (94–99% of the simulation), forming strong hydrogen bonds. This constant connection makes ASP86 the main “anchor” for YCWS throughout the 100-nanosecond simulation. Other important residues, Gln130 and Glu81, formed hydrogen bonds, staying connected 63% and 40% of the time, respectively. Lys33 and Asn144 further stabilize the complex through hydrogen bonds and electrostatic interactions, connecting 54% and 31% of the time. Interestingly, Lys33 also interacted with a chloride ion (63% of the time), building a very strong network. This complex and frequent connection pattern highlights how specifically and strongly YCWS binds to the protein.

#### 3.2.2. Binding Free Energy Evaluation Using Prime-MMGBSA

The Prime MMGBSA analysis reveals that compound YCWS exhibited a higher negative binding energy of −78.0 kcal/mol, indicating stronger binding stability, followed by YWCS (−57.1 kcal/mol), DHYW (−39.10 kcal/mol), VWCS (−38.16 kcal/mol), and FWYS (−33.91 kcal/mol). These findings suggest that the YCWS complex has superior binding stability, further supporting its identification as a potential lead molecule.

### 3.3. In Vitro Analysis of Peptide YCWS as an Inhibitor of Cdk5

On the basis of a molecular modeling study, YCWS was found to have the most efficient binding capacity with the Cdk5 protein. Hence, further in vitro analysis was performed with the peptide YCWS.

#### 3.3.1. Binding Study of Peptide by SPR

SPR analysis showed the real-time biomolecular interactions between the peptide and the immobilized Cdk5 protein on the Ni-NTA chip. Significant changes in RU were observed on passing the increasing concentration of peptide over the protein. The KA and KD values of the peptide were found to be 2.54 × 10^8^ M and 3.94 × 10^−9^ M, respectively. The KD value of YCWS illustrated a higher strength of interaction with the protein (Figure 5D).

#### 3.3.2. Cell Cytotoxicity Assay

**Figure 6:**
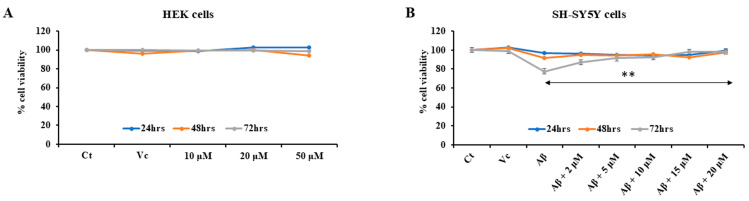
Cytotoxicity and rescue effect of peptide on cells treated with three different concentrations, i.e., 10 µM, 20 µM and 50 µM for 24 h, 48 h and 72 h: (**A**) HEK-293 cells (**B**) SH-SH5Y cells. Experiments were performed in triplicate and data is represented as mean ± SD. Control (Ct), vehicle control (Vc) and amyloid beta (Aβ). The *p*-value is calculated between the Aβ-treated group and the Aβ + 20 µM peptide-treated group using the Mann–Whitney test. ** *p* < 0.001.

The cell cytotoxicity assay was performed with the HEK-293 cell line, using the MTT reduction assay to assess the effect of the peptide on normal cells. The peptide YCWS was found to be nontoxic on the HEK-293 cells at 24 h, 48 h, and 72 h up to 50 µM concentration (Figure 6A).

#### 3.3.3. Neurotoxic Rescue Effect of YCWS in SH-SY5Y Cells

It has been reported that Aβ-amyloid plays an important role in the development of AD, and cortical neurons undergo death after exposure to Aβ-amyloid in vitro. Aggregated Aβ_25–35_ was able to induce a significant neuronal death at a concentration of 20 µM. Retinoic acid results in differentiation of SH-SY5Y cells from an initial epithelial-like cell phenotype into a more expansive and branched neuronal phenotype. The peptide, YCWS, showed rescue against neuronal death in a dose-dependent manner and increased cell viability. The aggregated Aβ_25–35_ toxicity results in 77.65% viability in SH-SY5Y cells as compared to the control groups. Peptide treatment increases the cell viability to 87.28%, 91.47%, 92.20%, 98.27% and up to 98.55% with 2 µM, 5 µM, 10 µM, 15 µM, and 20 µM concentrations, respectively (Figure 6B).

#### 3.3.4. Expression of Cdk5, Mcl1, Aβ-Amyloid, Tau, and *p*-Tau Proteins by the Treatment of YCWS in SH-SY5Y Cells

Aβ-amyloid deposition and hyperphosphorylation of tau protein are the prime suspects responsible for the disease pathology. Cdk5 is known to phosphorylate tau protein, causing it to dissociate from microtubules, self-aggregate, and eventually form hyperphosphorylated tau and insoluble neurofibrillary tangles. Studies have demonstrated that inhibiting Cdk5 in vitro can reduce tau hyperphosphorylation and NFT formation. Inhibition of Cdk5 thus helps in targeting abnormal tau, specifically its hyperphosphorylated form and Aβ-amyloid accumulation. The YCWS treatment results in the inhibition of Cdk5 in a dose-dependent manner. The expression of Cdk5 was reduced to 75.99% in the treated cells. This shows that the peptide YCWS has the potential role of inhibiting the protein kinase activity.

This result was further validated by RT-PCR; mRNA from all six groups was subjected to RT-PCR using specific primers for Cdk5. There was a 1.85-fold increase in the expression of Cdk5 mRNA in aggregated Aβ_25–35_-treated cells as compared to the control group, and on treatment with the different concentrations of YCWS, the expression decreases to 1.34-fold, 1.16-fold and 1.08-fold in the 2 µM, 5 µM and 10 µM doses of peptide, respectively, as compared to the control group.

Deregulated Cdk5 phosphorylates Mcl1, triggering its degradation by ubiquitylation, which results in mitochondrial dysfunction and subsequent neuronal death. The peptide treatment results in inhibition of Cdk5, which further upregulates the expression of Mcl1 in the treatment groups. The expression of Mcl1 was found to be 22.43% more in the treated group. It has been reported that enhancing Mcl1 levels offers comparable neuroprotection, as Mcl1 degradation is a key mechanism by which Cdk5 promotes neurotoxicity in AD. Thus, the upregulation of Mcl1 via inhibition of Cdk5 can be a potential therapeutic strategy for delaying or preventing neurodegeneration in AD.

**Figure 7:**
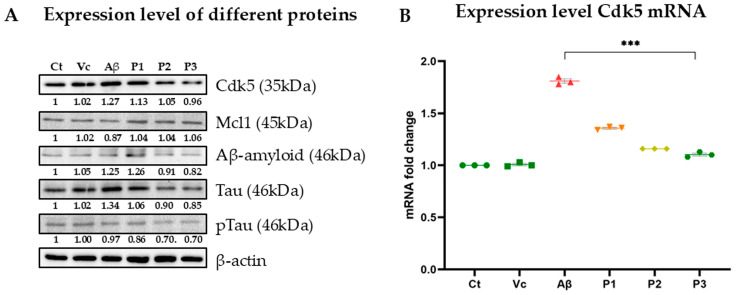
Effect of peptide YCWS on the expression level of (**A**) various proteins (Cdk5, Mcl1, Aβ-amyloid, Tau, and pTau) in different experimental groups of the Aβ-treated SH-SY5Y cell line treated with three different concentrations of peptide P1 (2 µM), P2 (5 µM) and P3 (10 µM) in culture medium for 72 h at 37 °C. (**B**) Cdk5 mRNA in all group’s was further validated by RT-PCR. Experiments were performed in triplicate and data is represented as mean ± SD (protein level) and mean ± SEM (mRNA level). The *p*-value is calculated between the Aβ-treated group and the Aβ + 10 µM peptide-treated group using the Mann–Whitney test. *** *p*-value ≤ 0.0001.

The expression level of the major hallmarks of the disease, i.e., Aβ-amyloid, tau, and *p*-Tau, was also found to be increased in the aggregated Aβ_25–35_-treated groups, and their expression level decreases with the increasing concentration of peptide by 67.42%, 63.56% and 72.63%, respectively. This shows that the peptide was able to rescue the neurotoxicity caused by aggregated Aβ_25–35_ (Figure 7).

## 4. Discussion

AD is primarily characterized by memory impairment and a decline in overall cognitive functions. This neurodegenerative condition can begin silently, with subtle changes in brain structure and function occurring up to two decades before symptoms manifest. Only after years of these underlying changes do individuals start to experience noticeable cognitive difficulties. Cdk5 plays a critical role in higher cognitive functions, including synaptic plasticity, learning, and memory formation [39]. Dysregulation of Cdk5 activity, either through hyperactivation or reduction, can contribute to neurodegenerative processes.

A total of 659 individuals visited the Memory Clinic of the Department of Geriatric Medicine, AIIMS, New Delhi. Out of which, 61 subjects were diagnosed with AD and 55 diagnosed with MCI via different clinical assessments with low HMSE and ACEIII scores and on the basis of neuroimaging MRI, tau PET, FDG PET, and routine laboratory tests. Also, 57 individuals were recruited from the Geriatric Medicine OPD as GC subjects to compare their protein concentration in blood samples with AD and MCI subjects.

Age is the biggest known risk factor for AD, and the National Institute on Aging has stated that people over the age of 65 are most likely to have AD. About one in 13 people, 65–84 y and one in three people 85 y and older live with Alzheimer’s [40]. In our study, we have recruited the dementia patients above 60 y and classified them in 3 age groups, i.e., 60–65 y, 66–75 y, and ≥76 y. The maximum AD (62.30%) and MCI (69.09%) patients were found to be in the above 65 age group, i.e., in between 66 and 75 y.

Though it is stated in the literature that illiteracy is common in AD, in our study group, more than 80% of AD patients were literate. In India, mainly in rural societies, there is a significant stigma associated with dementia. This can discourage people from seeking help, especially if they are illiterate, as they face additional barriers to accessing healthcare. Illiterate individuals may not be aware of the symptoms of AD or may attribute them to normal aging. These results also matched our results of a higher percentage of AD in the urban population (63.93%) compared to the rural population (36.07%). AD can be caused by inheritance from family or by sporadic factors. It has been reported that only 1% of AD cases are found to be hereditary [41]. Our study group also showed that more than 77% of AD cases and 74% of MCI patients do not have a family history.

Accumulation of reactive oxygen species (ROS) due to various environmental factors, including injury, chronic inflammation, aging, and oxidative stress, contributes to neurotoxic insults. These insults lead to an elevated calpain activity, which in turn upregulates p35 and p39, two activators of Cdk5 [11,42]. The subsequent hyperactivation of Cdk5 results in the aberrant phosphorylation of Tau and proteolytic cleavage of amyloid precursor protein (APP), promoting neurodegeneration [43]. Additionally, Cdk5-mediated phosphorylation of Mcl1 induces its degradation, impairing neuronal autophagy and further exacerbating neurotoxicity. Mcl1 is the direct specific substrate of Cdk5, which is not shared by any other kinases, making it different from others [44]. A proteomic analysis by Olah et al. (2015) revealed elevated Cdk5 levels in the cerebrospinal fluid (CSF) of AD patients [45]. To investigate the systemic implications of Cdk5 dysregulation, the current study examined the Cdk5 and Mcl1 levels in the blood of AD, MCI, and age-matched control subjects. The findings demonstrated significantly higher Cdk5 levels in AD patients compared to both the MCI and control groups. Moreover, MCI patients exhibited elevated Cdk5 levels compared to controls. These results corroborate previous findings by Sultana et al. (2007), who reported increased Cdk5 expression in the hippocampus of MCI patients, suggesting that Cdk5 dysregulation may be an early event in the progression of dementia [46].

Our study further demonstrated a significant age-dependent increase in Cdk5 levels in AD patients. In subsequent analyses, we have observed significantly decreased Mcl1 protein levels in AD patients compared to both MCI patients and healthy controls. In the earlier study by Kumar Nikhil Shah et al., 2017, the level of Mcl1 was also found to be decreased in the brain tissue of AD patients as compared to the normal controls [19].

These findings were further supported by quantifying the mRNA expression levels of Cdk5 and Mcl1 in each study group. Compared to MCI and control groups, AD patients exhibited a significant upregulation (2.25-fold) of Cdk5 mRNA. Conversely, a significant (0.54-fold) downregulation of Mcl1 mRNA was observed in AD patients relative to both MCI and control groups. It has been previously reported that aberrant Cdk5 hyperactivation leads to phosphorylation of Mcl1 at T92, which initiates Mcl1 ubiquitination and subsequent proteasomal degradation. This process is associated with mitochondrial dysfunction, neuronal cell death, and the progression of AD pathology [19,47].

The level of *p*-Tau protein of the same patient was also found to be elevated in serum, which matches the evidence that the overactivation is associated with hyperphosphorylation of Tau. Our observation of elevated *p*-Tau in the serum of the same patients with elevated levels of Cdk5 aligns with previous evidence linking overactivation to tau hyperphosphorylation [11,48,49,50,51,52,53,54,55]. Blood Cdk5 levels, as determined by ROC analysis, can effectively differentiate between AD, MCI, and GC, demonstrating significant differences with high sensitivity, specificity, and an AUC > 0.90. Similarly, decreased blood Mcl1 levels directly associated with Cdk5 can also significantly differentiate each disease group. Early diagnosis of AD may be facilitated by the identification of these biomarkers.

These findings suggest that Cdk5 and Mcl1 could serve as potential blood-based biomarkers for the diagnosis of AD and MCI. Given its pivotal role in AD pathogenesis, Cdk5 has emerged as a promising therapeutic target. Inhibiting Cdk5 activity could potentially mitigate the neurodegenerative processes associated with the disease [1,56]. Successively increasing Mcl1 level protects the mitochondrial dysfunction and thereby neuroprotection [19].

Numerous Cdk5 inhibitors have been reported to mitigate neurological disorders in mouse models [57,58], and some small peptides have been shown to inhibit the Cdk5–p25 complex, thereby protecting against neurodegeneration [59,60]. Amino pyrazole compounds have been demonstrated to prevent neurodegeneration by inhibiting Cdk5/p35 activity in the brain [61]. Some drugs targeting tau tangles by inhibiting Cdk5 are in the preclinical stage and clinical trials [62]. Inhibition of Cdk5 can also reduce the microglial expression of pro-inflammatory cytokines, which overexpress Aβ-amyloid [63]. The structure of Cdk5 has two lobes: the N-terminal glycine-rich loop (G-loop), called the inhibitory part, and the C-terminal T loop, containing the phosphorylated residues, serine, or threonine, making it the activation site. The catalytic site consists of the active binding site, Asp26, and the ATP binding site, Lys33 [28]. Glu51, Ala143, and Lys33 are the conserved residues, forming a catalytic triad [64]. Inhibition of Cdk5 by many synthetic inhibitors like roscovitine, olomoucin, and purvalanol-A is through the ATP binding site [65]. Roscovitine forms hydrogen bonds with Gln130, Asp 86, and Cys83 [28]. Many pyrazole compounds are also known to bind with Cdk5 at the ATP binding site, conserving their critical interaction between the piperidine group and Asp 144 along with Cys133 [66]. These inhibitors result in functional alteration of Cdk5, which further reduces the phosphorylated tau and improves various cognitive parameters.

Our in silico studies have shown that tetrapeptide-based ligands can effectively bind to the active site of Cdk5, suggesting their potential as inhibitors. The screening of these tetrapeptides by molecular docking followed by simulation studies revealed that YCWS offers the strongest binding capacity with Cdk5 (binding energy −78.0 kcal/mol) among others. It forms hydrogen bonds with Asp86, Asp84, Cys83, Asn144, Gln130, Lys33, Glu12, Ile10, and Asp92 at the ATP binding site of Cdk5 and several hydrophobic interactions with Ala31, Ala143, Ile10, Val16, Val18, Phe82, and Leu133. The compound exhibits a high affinity for the Cdk5 ATP-binding pocket, forming a greater number of hydrogen bonds and hydrophobic interactions than previously reported inhibitors. Additionally, it interacts with the catalytic triad, suggesting a unique binding mode that may contribute to its selectivity and potency. These ligands could offer a novel therapeutic strategy by directly targeting Cdk5 and modulating its activity. The short peptide inhibitor has a handful of advantages as a therapeutic agent, including having a short life, being highly specific and potent, being easily acceptable by the body and being non-toxic.

In silico and in vitro studies demonstrated that the peptide YCWS binds to Cdk5 with high affinity and specificity. The nanomolar binding affinity, quantified by a dissociation constant of 3.94 × 10^−9^ M, is significantly stronger than that of any other known Cdk5 inhibitor. The peptide YCWS was found to be non-toxic in normal HEK cells as well as in SHSY-5Y cells. The induction of aggregated Aβ_25–35_ increased the expression of Cdk5 and thereby increased Aβ-amyloid, Tau, and *p*-Tau levels in the SH-SY5Y AD cell line. The treatment of peptide YCWS in the AD cell line rescued neurotoxicity by increasing the level of Mcl1, thus decreasing the expression level of Cdk5 as well as Tau, *p*-Tau, and Aβ-amyloid. The protein Mcl1 maintains the autophagy of neurons and also regulates apoptosis via the release of cytochrome c from mitochondria, which initiates cell death in neuronal cells [67]. It also agitates microglial cells, resulting in the release of more and more cytokines. The suppression of overexpressed Cdk5 can control the hyperphosphorylation of many substrates, which causes mitochondrial dysfunction. Therefore, restricting Cdk5 expression under neurotoxic conditions presents a promising therapeutic strategy to delay or prevent AD progression. Further, this study identifies Cdk5 as a promising blood-based diagnostic biomarker of AD and MCI.

## 5. Conclusions

Cdk5, a crucial and unique Cyclin-Dependent Kinase, is found to be highly expressed in the blood of AD and MCI as compared to GC. Hyperactivation of Cdk5 during diseased conditions results in upregulation of major disease hallmarks, i.e., Aβ-amyloid, Tau, and pTau, and downregulation of Mcl1, as observed in the AD cellular model. Also, inhibition of Cdk5 by a peptide inhibitor, YCWS, rescued neurotoxicity in the cellular model. This study first reported the Cdk5 and Mcl1 in the blood levels of AD and MCI and compared them with GC. Our results strongly emphasize that Cdk5 can be considered as a potential blood-based biomarker and attractive therapeutic target for delaying and perhaps preventing neurodegeneration in AD patients.

### Limitations of the Study

The ROC-derived cutoffs for Cdk5 and Mcl1 demonstrate high sensitivity and specificity within our study cohort; these findings may be subject to overfitting due to the absence of external validation.

## Data Availability

All data generated or analyzed during this study are included in this published article and its Supplementary Information Files.

## Supplementary Materials

The following supporting information can be downloaded at https://www.mdpi.com/article/10.3390/biom15101365/s1. Figure S1: Characterization of pure proteins and standard curves; Figure S2: Validation Cdk5 and Mcl1 in serum level by Western blot; Figure S3: Ligand-interaction diagram of tetrapeptides showing hydrogen bonding (magenta line) with Cdk5 (PDB: IUNL) protein. (A) YWCS (B) VWCS (C) DHYW (D) FWYS; Figure S4: Binding pattern of YCWS with Cdk5; Figure S5: Binding of YCWS with Cdk5; Table S1: Demographic and Clinical data of AD, MCI and GC subjects; Table S2: The serum concentration of Cdk5 (ng/μL) shown as mean ± SD with different attributes; Table S3: The serum concentration of Mcl1 (ng/μL) shown as mean ± SD with different attributes; Table S4: Comparison of Area under curve, Sensitivity, Specificity, Sen + Spe, Youden Index, and *p* value of Cdk5 and Mcl1 blood levels in between study groups (AD, MCI and GC).
