# Supplementary material for: Targeting a Tau Kinase Cdk5, Cyclin-Dependent Kinase: A Blood-Based Diagnostic Marker and Therapeutic Earmark for Alzheimer’s Disease
Source: Biomolecules. 2025 Sep 26;15(10):1365. doi: 10.3390/biom15101365 (PMC12562681; doi:10.3390/biom15101365)
Supplement: Supplementary file 1 [file biomolecules-15-01365-s001.zip › biomolecules-3791381-2.pdf]

Western blot for serum sample

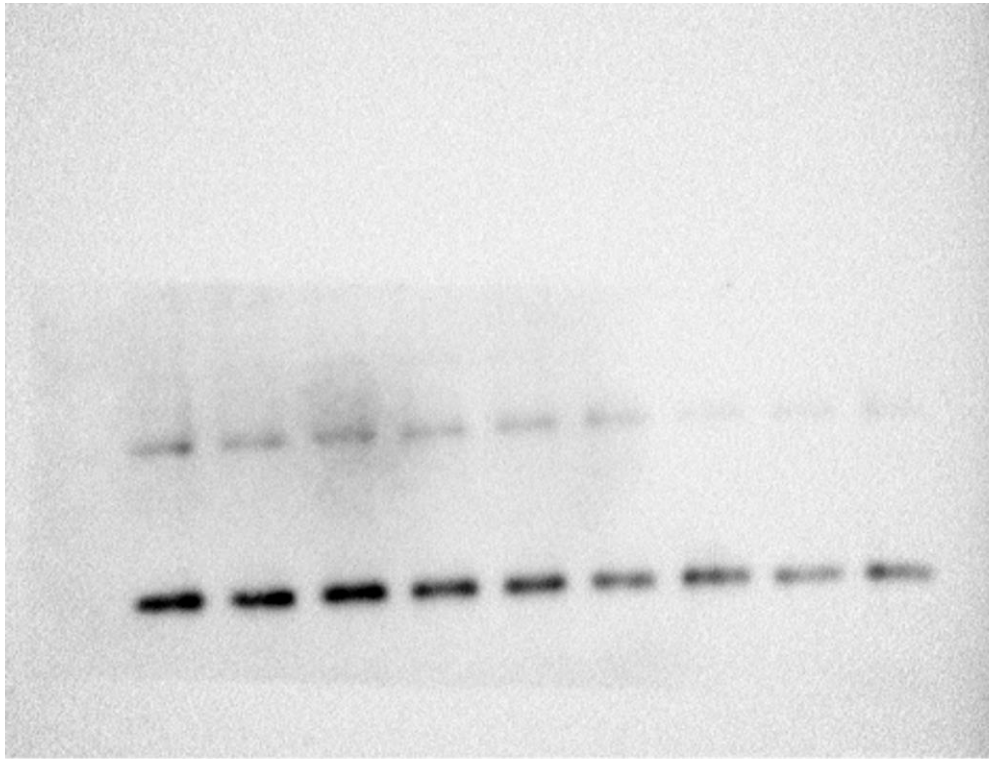

CDK-5

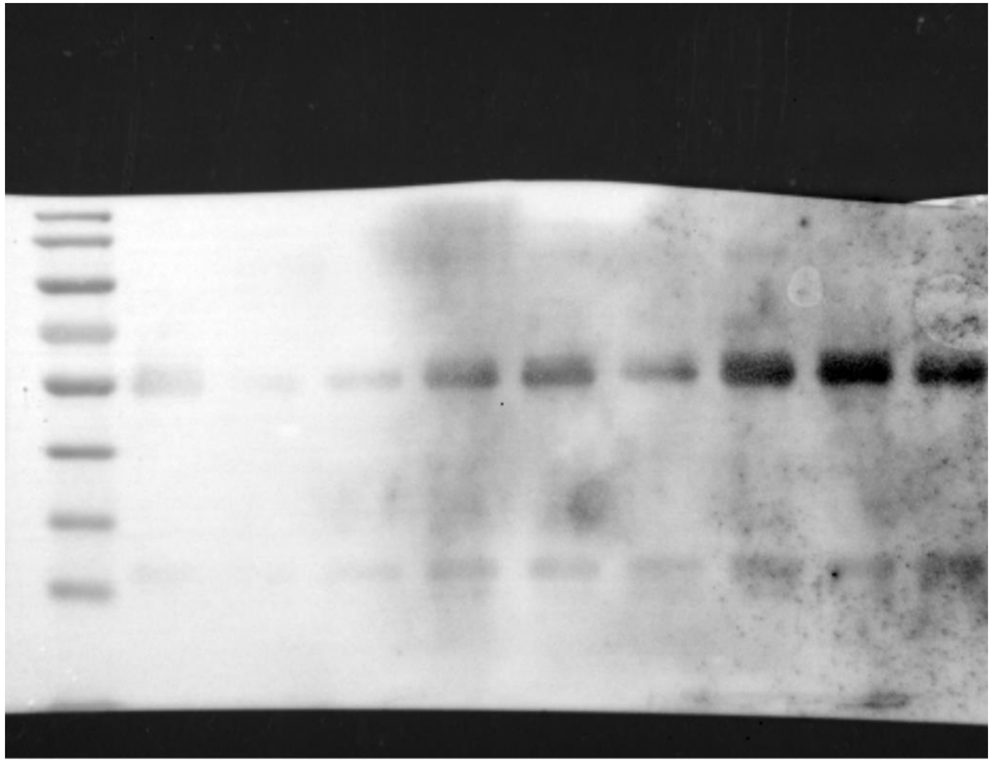

MCL-1

Western blot for cell lysate

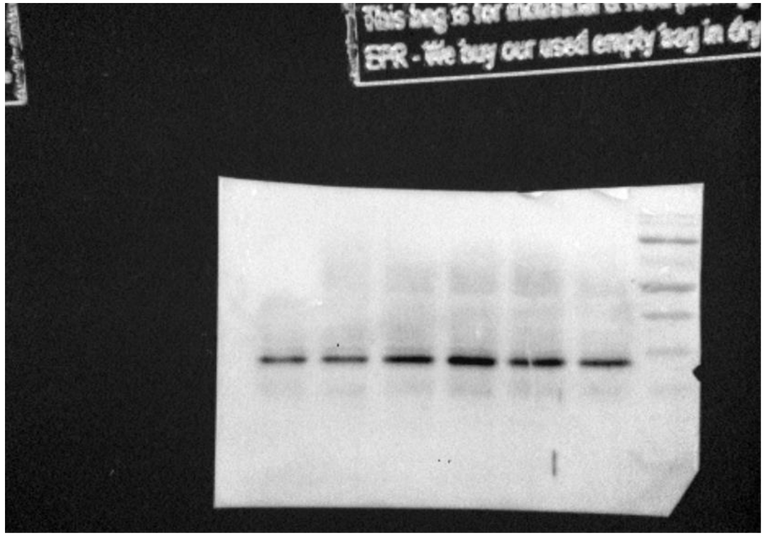

Tau

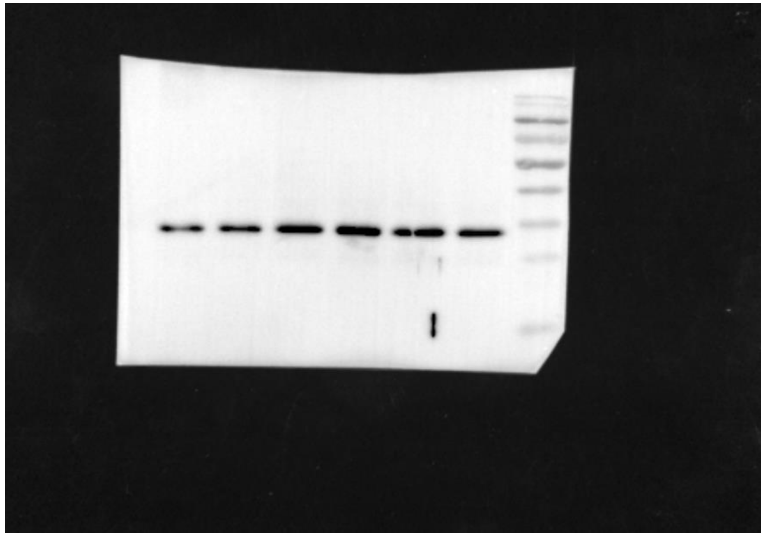

Cdk5

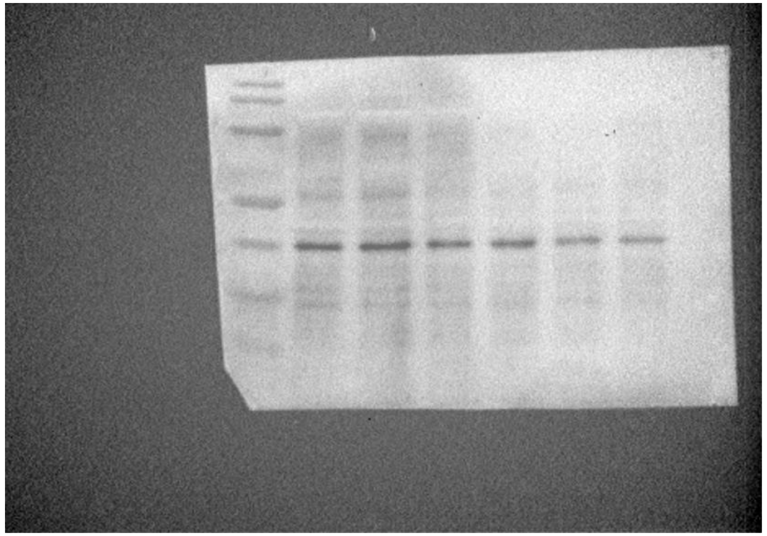

pTau

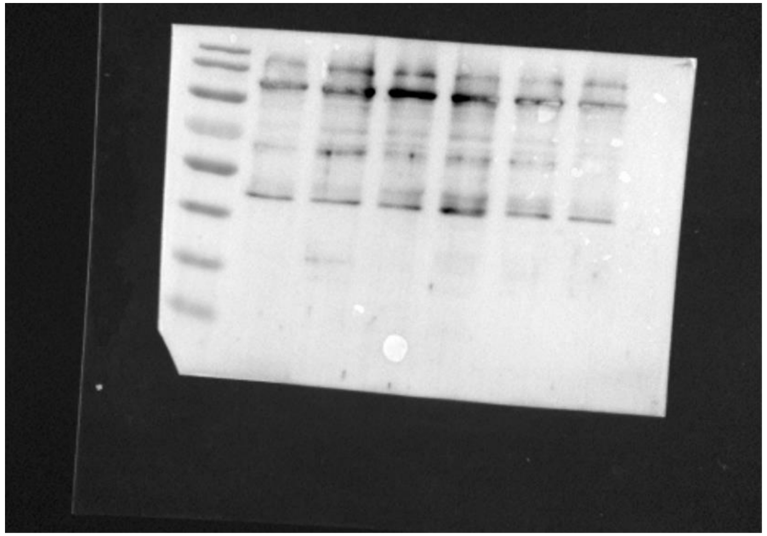

Amyloid beta

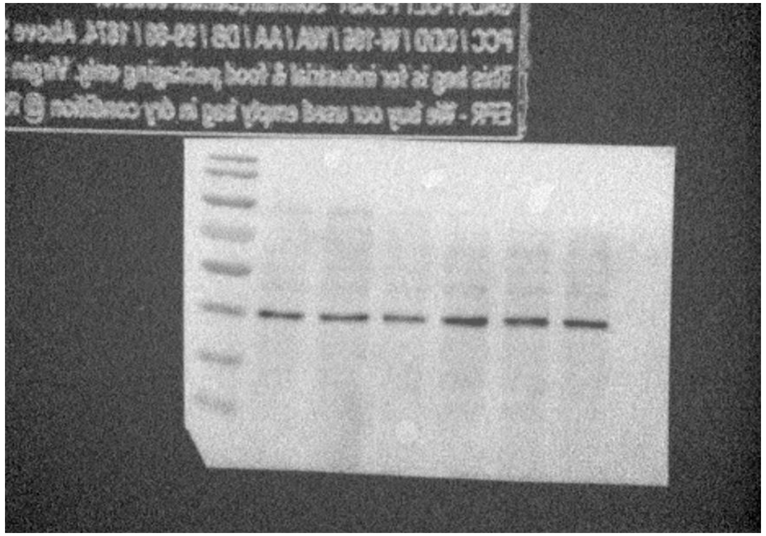

MCL-1

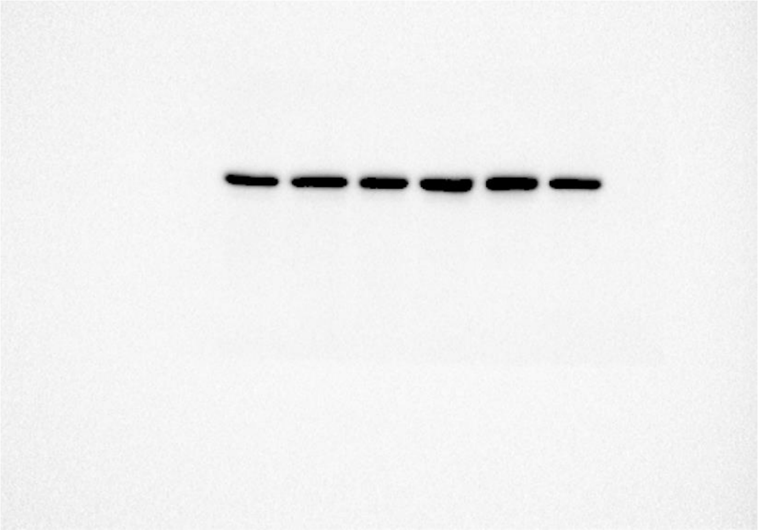

B actin
