# Supplementary material for: Targeting a Tau Kinase Cdk5, Cyclin-Dependent Kinase: A Blood-Based Diagnostic Marker and Therapeutic Earmark for Alzheimer’s Disease
Source: Biomolecules. 2025 Sep 26;15(10):1365. doi: 10.3390/biom15101365 (PMC12562681; doi:10.3390/biom15101365)
Supplement: Supplementary file 1 [file biomolecules-15-01365-s001.zip › Supplementary Tables S1-S4.pdf]

Supplementary Table S1: Demographic and Clinical data of AD, MCI and GC subjects

**Parameters**	**AD**	**MCI**	**GC**	**p value**
**N**	61	55	57	
**Sex**, n (%)				
Male	34 (55.74%)	33 (60.00%)	36 (63.16%)	0.712
Female	27 (44.26%)	22 (40.00%)	21 (36.84%)	
**Age category**, n (%)				
60-65 Yrs	5 (8.20%)	5 (9.09%)	16 (28.07%)	0.009
66-75 Yrs	38 (62.30%)	38 (69.09%)	33 (57.89%)	
≥76 Yrs	18 (29.51%)	12 (21.82%)	8 (14.04%)	
**Age** (mean ± SD)	73.88 ± 6.89	71.56 ± 4.70	69.71 ± 6.03	0.0010
**Education**, n (%)				
Illiterate	11 (18.03%)	10 (18.18%)	16 (28.07%)	0.323
Literate	50 (81.97%)	45 (81.82%)	41 (71.93%)	
**Occupation**, n (%)				
Unemployed	23 (37.70%)	18 (32.73%)	18 (31.58%)	0.712
Employed (Farming, business)	16 (26.23%)	18 (32.73%)	22 (38.60%)	
Retired	22 (36.07%)	19 (34.55%)	17 (29.82%)	
**Locality, n (%)**				
Urban	39 (63.93%)	38 (69.09%)	38 (66.67%)	0.841
Rural	22 (36.07%)	17 (30.91%)	19 (33.33%)	
**Family history of AD**, n (%)				
Yes	14 (22.95%)	14 (25.45%)	5 (8.77%)	0.051
No	47 (77.05%)	41 (74.55%)	52 (91.23%)	
**Duration of disease (Years)**, n (%)				
Nil	0 (0.00%)	0 (0.00%)	57 (100.00%)	0.000
Upto 2 Years	19 (31.15%)	20 (36.36%)	0 (0.00%)	
Above 2 Years	42 (68.85%)	35 (63.64%)	0 (0.00%)	
**CDI** (mean ± SD)	59.06 ± 28.36	23.21 ± 23.53	6.88 ± 19.96	0.000
**HMSE** (mean ± SD)	12.29 ± 5.55	20.92 ± 4.65	28.10 ± 1.73	0.000
**ACE III** (mean ± SD)	37.77 ± 18.28	64.36 ± 10.20	91.85 ± 4.82	0.000
**GDS** (mean ± SD)	3.59 ± 3.08	3.54 ± 3.29	2.10 ± 2.67	0.0128
**Dm**, n (%)				
Yes	22 (36.07%)	15 (27.27%)	8 (14.04%)	0.024
No	39 (63.93%)	40 (72.73%)	49 (85.96%)	
**Htn**, n (%)				
Yes	35 (57.38%)	25 (45.45%)	26 (45.61%)	0.330
No	26 (42.62%)	30 (54.55%)	31 (54.39%)	

HMSE -Scores <26 out of 30 and ACE III Scores <72 is cognitively impaired. GDS- Scores >9 out of 15 is suggestive of depression. CDI- Cognitive Disability Index with higher scores indicating increased disability and functionality impairment. p values are based on one way ANOVA for continuous variables and chi-square tests for categorical variables.

Supplementary Table S2: The serum concentration of Cdk5 (ng/µl) shown as mean ± SD with different attributes.

Cdk5 Conc. (ng/ul)	AD (N=61)	MCI (N=55)	GC (N=57)
**Sex**			
Male	29.21 ± 4.76	26.16 ± 2.29	22.57 ± 1.92
Female	29.68 ± 6.46	26.21 ± 3.52	22.44 ± 2.86
p value	0.6277	0.5237	0.4215
**Age**			
≤60-65 Yrs	21.98 ± 1.00	24.57 ± 2.30	22.85 ± 2.16
66-75 Yrs	28.76 ± 4.11	26.20 ± 2.57	22.22 ± 2.47
≥76 Yrs	32.88 ± 6.41	29.89 ± 2.54	23.09 ± 1.67
p value	0.0001	0.0010	0.5119
**Education**			
Illiterate	28.80 ± 7.28	27.38 ± 3.34	23.72 ± 2.02
Literate	29.56 ± 5.15	25.91 ± 2.65	22.05 ± 2.23
p value	0.6574	0.0688	0.0061
**Locality**			
Urban	29.26 ± 5.47	25.92 ± 2.94	22.53 ± 2.26
Rural	29.70 ± 5.77	26.75 ± 2.50	22.51 ± 2.39
p value	0.6149	0.8406	0.4882
**Family History Of AD**			
No	29.68 ± 5.35	26.60 ± 3.06	22.49 ± 2.31
Yes	28.55 ± 6.24	24.96 ± 1.41	22.87 ± 2.24
p value	0.2544	0.0302	0.6372
**Disease Duration**			
**Nil**	-	-	22.52 ± 2.28
Upto 2 Years	28.85 ± 5.13	26.42 ± 2.85	-
>2 Years	29.68 ± 5.75	26.05 ± 2.82	-
p value	0.7029	0.3217	-
**Htn**			
No	29.30 ± 5.92	25.37 ± 2.18	22.36 ± 2.66
Yes	29.51 ± 5.31	27.15 ± 3.20	22.71 ± 1.76
p value	0.5569	0.9909	0.7184
**Dm**			
No	30.35 ± 6.11	26.07 ± 2.88	22.55 ± 2.38
Yes	27.77 ± 3.93	26.46 ± 2.69	22.34 ± 1.65
p value	0.0404	0.6739	0.4080

Supplementary Table S3: The serum concentration of Mcl1 (ng/µl) shown as mean ± SD with different attributes.

Mcl1 Conc. (ng/µl)	AD (N=61)	MCI (N=55)	GC (N=57)
**Sex**			
Male	19.22 ± 3.41	23.14 ± 1.28	26.01 ± 2.26
Female	19.85 ± 3.57	23.02 ± 1.27	25.26 ± 1.42
p value	0.7579	0.3718	0.0905
**Age**			
≤60-65 Yrs	25.39 ± 0.95	23.28 ± 1.07	25.78 ± 2.02
66-75 Yrs	19.19 ± 3.15	23.23 ± 1.22	25.71 ± 2.13
≥76 Yrs	18.53 ± 3.03	21.60 ± 1.32	25.73 ± 1.71
p value	0.0001	0.0190	0.9927
**Education**			
Illiterate	19.49 ± 2.87	23.23 ± 1.32	24.94 ± 1.61
Literate	19.50 ± 3.61	23.06 ± 1.27	26.04 ± 2.09
p value	0.5040	0.3583	0.9681
**Locality**			
Urban	19.63 ± 3.49	23.05 ± 1.18	25.73 ± 1.54
Rural	19.28 ± 3.50	23.20 ± 1.48	25.74 ± 2.78
p value	0.3550	0.6572	0.5104
**Family History Of AD**			
No	19.31 ± 3.36	23.13 ± 1.37	25.78 ± 2.05
Yes	20.16 ± 3.87	22.97 ± 0.91	25.27 ± 1.70
p value	0.7891	0.3403	0.2979
**Disease Duration**			
Nil	-	-	25.73 ± 2.01
Upto 2 Years	19.77 ± 3.20	23.16 ± 1.37	-
Above 2 Years	19.38 ± 3.61	23.06 ± 1.22	-
p value	0.3456	0.3924	-
**Htn**			
No	19.35 ± 3.72	23.29 ± 1.09	25.91 ± 2.28
Yes	19.61 ± 3.32	22.85 ± 1.43	25.51 ± 1.65
p value	0.6110	0.1030	0.2304
**Dm**			
No	19.64 ± 3.66	23.20 ± 1.29	25.62 ± 2.01
Yes	19.26 ± 3.17	22.81 ± 1.20	26.40 ± 2.01
p value	0.3441	0.1573	0.8430

Supplementary Table S4: Comparison of Area under curve, Sensitivity, Specificity, Sen + Spe, Youden Index, and p value of Cdk5 and Mcl1 blood levels in between study groups (AD, MCI and GC).

Serum Proteins	Area under curve	Cut-off	Sensitivity (Sen)	Specificity (Spe)	Sen + Spe	Youden Index	*p* value
**Cdk5 (AD vs GC)**	0.9057	24.97	89.66% (0.8966)	80.65% (0.8065)	1.7031	0.70	<0.0001
**Cdk5 (MCI vs GC)**	0.8516	24.26	79.31% (0.7931)	71.31% (0.7131)	1.5062	0.50	<0.0001
**Cdk5 (AD vs MCI)**	0.6737	26.75	66.07% (0.6607)	56.45% (0.5645)	1.2252	0.22	<0.0001
**Mcl1 (AD vs GC)**	0.9438	23.08	93.10% (0.9310)	85.48% (0.8548)	1.7858	0.78	<0.0001
**Mcl1 (MCI vs GC)**	0.8818	24.04	87.93% (0.8793)	80.36% (0.8036)	1.6829	0.68	<0.0001
**Mcl1 (AD vs MCI)**	0.8255	22.04	83.93% (0.8393)	75.81% (0.7581)	1.5974	0.59	<0.0001
