# Supplementary figures and images for: Targeting a Tau Kinase Cdk5, Cyclin-Dependent Kinase: A Blood-Based Diagnostic Marker and Therapeutic Earmark for Alzheimer’s Disease
Source: Biomolecules. 2025 Sep 26;15(10):1365. doi: 10.3390/biom15101365 (PMC12562681; doi:10.3390/biom15101365)

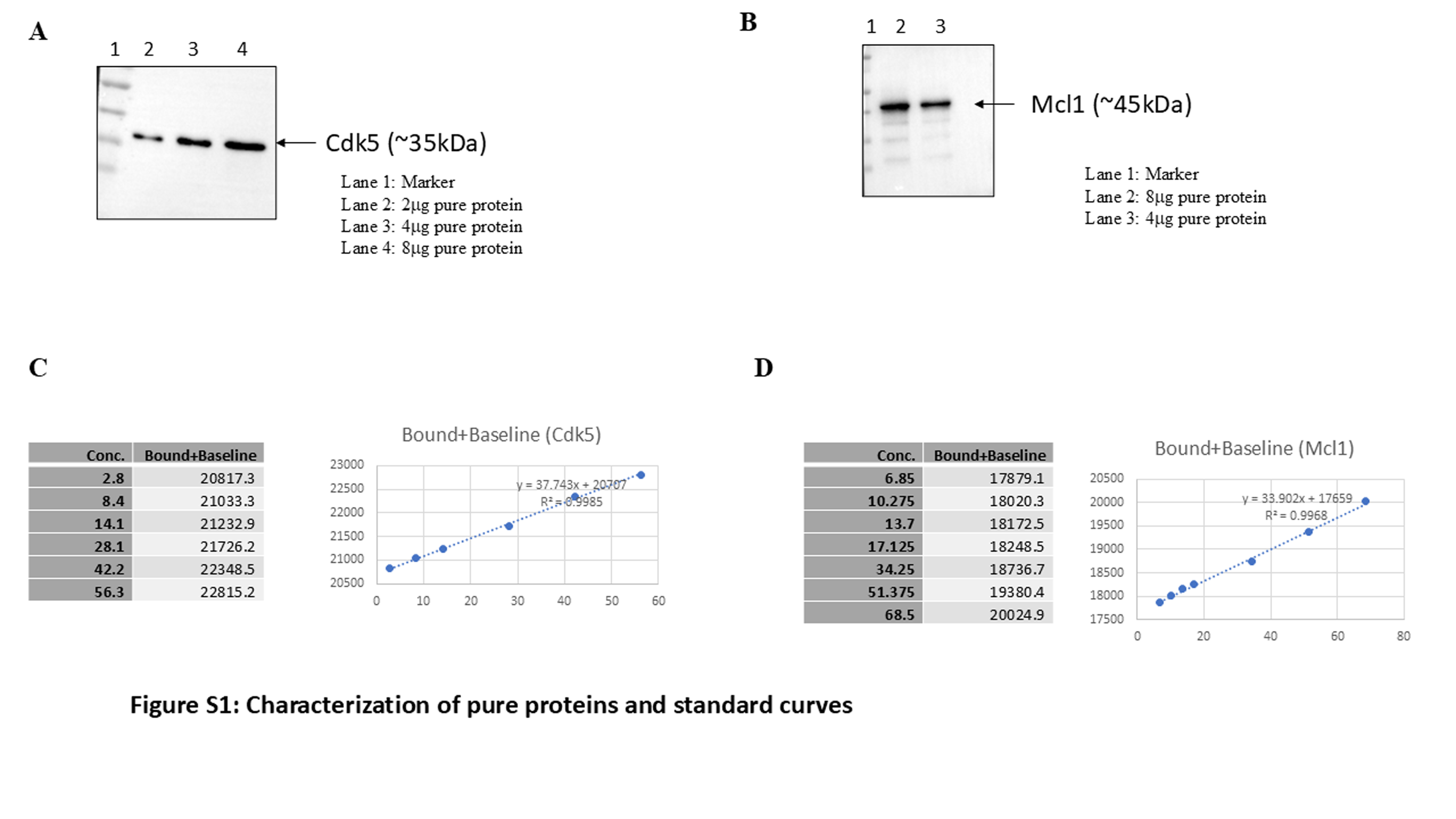

Supplement: Supplementary file 1 [file biomolecules-15-01365-s001.zip › Supplementary S1.tif]

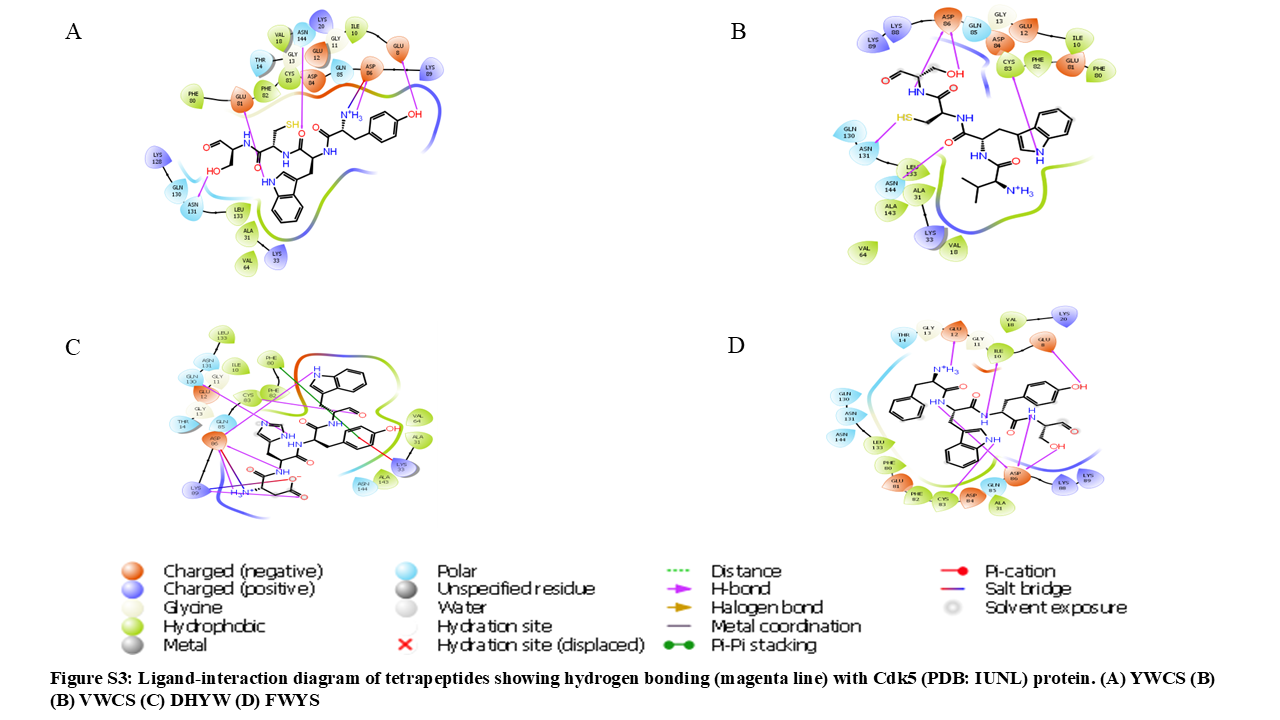

Supplement: Supplementary file 1 [file biomolecules-15-01365-s001.zip › supplementary S3.tif]

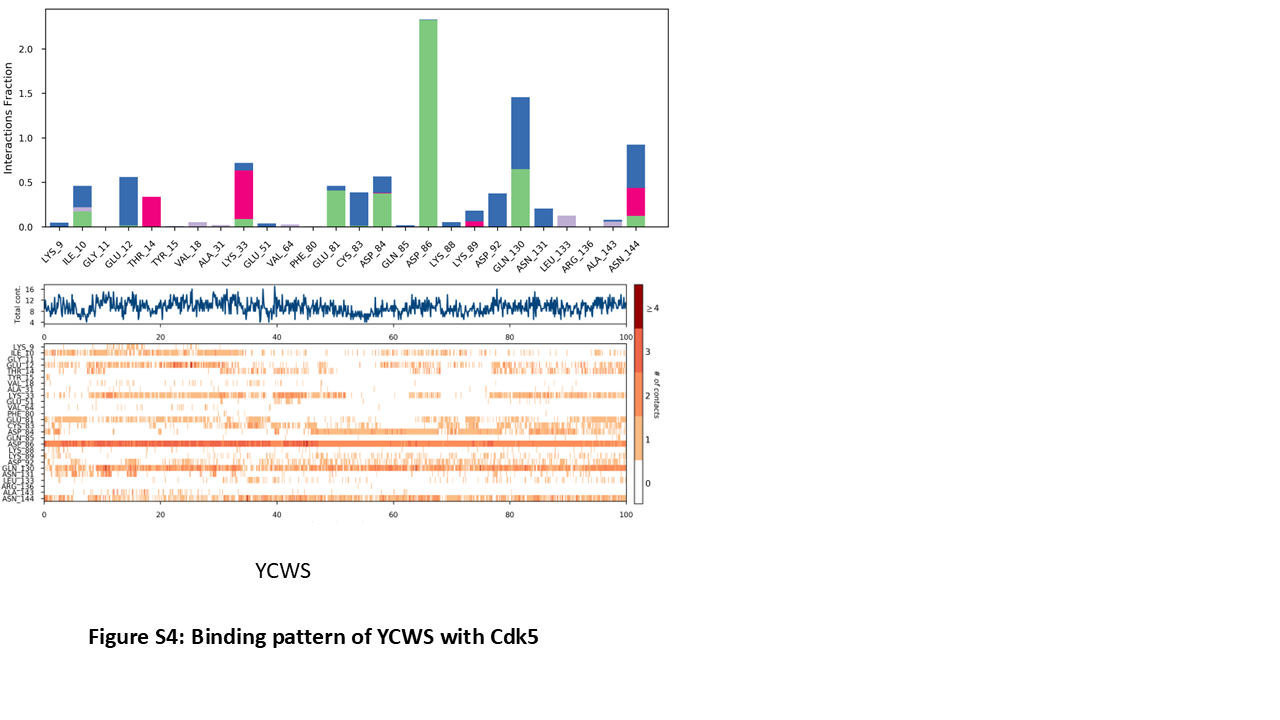

Supplement: Supplementary file 1 [file biomolecules-15-01365-s001.zip › Supplementary S4.tif]

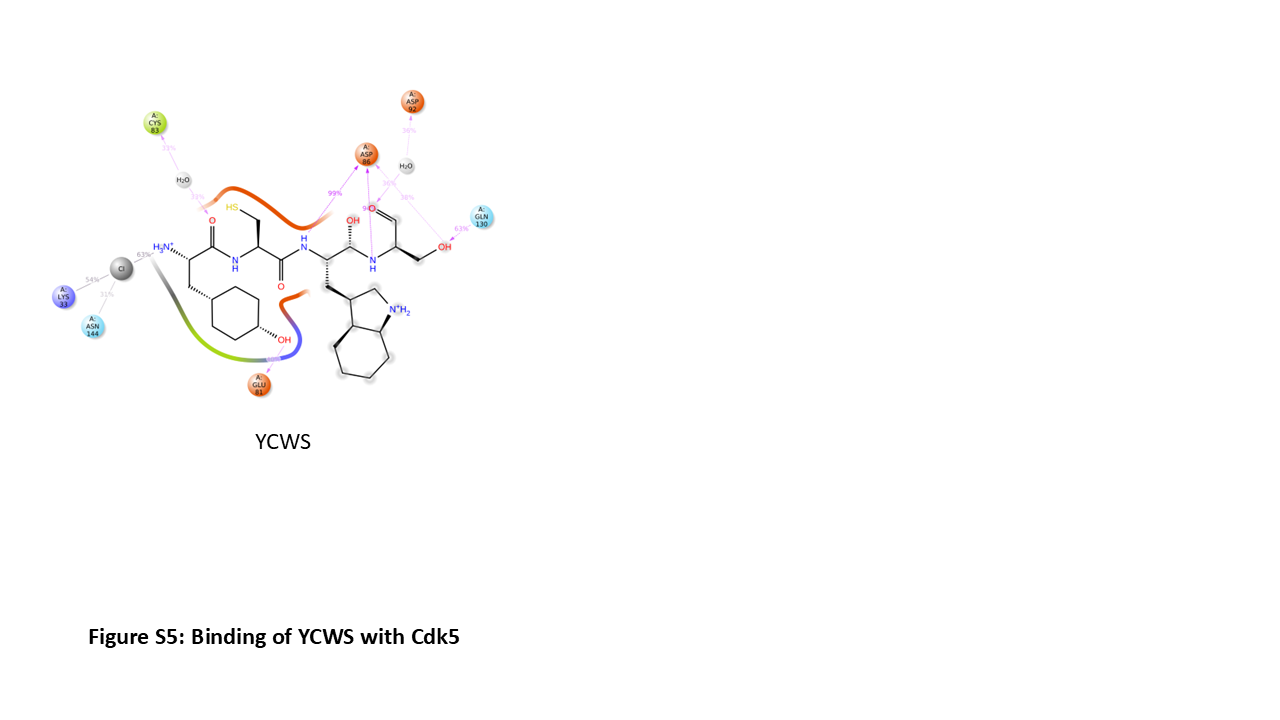

Supplement: Supplementary file 1 [file biomolecules-15-01365-s001.zip › Supplementary S5.tif]

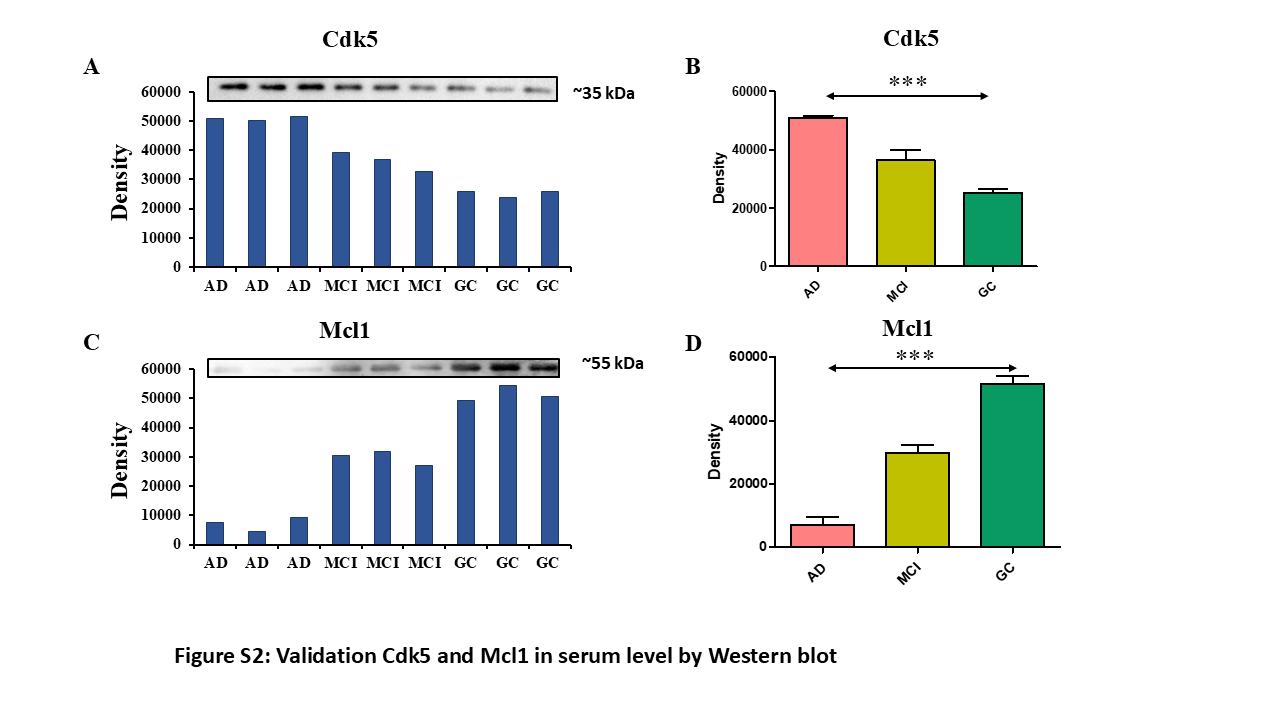

Supplement: Supplementary file 1 [file biomolecules-15-01365-s001.zip › supplementray S2.tif]
